# Editorial

**Published:** 1872-10

**Authors:** 


### EDITORIAL.

### Deterioration of Pulpless Teeth.

It is a fact generally recognized by every dentist of much experience or close observation, that the integrity of the enamel and dentine of pulpless teeth passes away; with however, varying degrees of rapidity, dependent upon the character of their structure; and this being modified by the original type, together with the condition and force of the organizing and developing agency at the time of their formation.

Usually teeth deprived of their pulps become in a comparatively short time friable, which is clearly shown by the readiness and frequency with which thin portions and attenuated edges break away. This is the difficulty that usually follows the operation of filling such teeth. It is, however, somewhat overcome or lessened since the introduction and use of heavy foils. By this means such feeble parts may be covered and protected far more effectually than when only thin foils were used. This impairment of the enamel and dentine occurs, because of the deprivation of nutrient supply; and it is not only exhibited in the manner referred to, but also by the increased susceptibility of the tooth substance to the action of decay-producing agents. Teeth vary exceedingly in the character of their structure; they have general features and aspects that are common to all, yet there is to every one an individuality which is constituted by variation in structural details. This variation is palpable to the unaided vision in respect to form and size, but is quite as plainly marked in the minute structure, under the microscope. These variations are, in the teeth, as in other structures or tissues, the occasion of every-varying susceptibilities, both in the living and the dead.

The organization in some is so thorough, and the dentine and enamel so nearly perfect, as to resist any ordinary decay-inducing agents that are brought into contact with them, and this sometimes to a great extent even after devitalization; while in other cases so inferior are these structures that they are scarcely able to maintain their integrity when but slightly and only for a short time exposed to the feeblest decomposing agents; even when possessed of their normal vitality, and with such, devitalization is followed by rapid and entire dissolution. Now there is an almost infinite variety of predispositions and susceptibilities between the extremes just presented; and the ability to apprehend and understand these conditions and susceptibilities, and in treatment to follow the indications is an attainment of no mean order, and one that should be sought by every dentist. The integrity of organized living tissue is maintained by the presence of the vital principle, together with the processes carried on by it, and as is already intimated the hold of the vitality upon the material part of tissues is exceedingly variable, in some holding a very firm grasp, and in others yielding its tenure at the first approach of danger. These things being true, the conclusion is forced upon us that the most acute and cultivated perception is requisite for the proper management of the teeth after they are attacked by disease. Teeth, the pulps of which are destroyed, have no vitality and receive no nutrition, and in common with all organized structures deprived of vitality immediately begin to undergo deterioration, and sooner or later suffer entire dissolution. With the teeth, however, this change may be materially modified--not prevented —by proper management, which consists in maintaining the most favorable conditions of the parts and tissnes round about them, and excluding so far as possible all injurious agents, and protecting the tooth at all points so far as may be from mechanical violence, either in the way of strokes or undue attrition. By attention, observation, and thought upon this subject, the educated dentist will not fail to perceive that pulpless teeth require the utmost care for their preservation for any considerable length of time.

That the conservation of living teeth is far easier than those devitalized.

And that the preservation of the pulps of the teeth is a matter of the utmost importance to those who desire to preserve their teeth in the best condition for the longest possible period.

### Peculiar Case.

Iowa, Sept. 30, 1782.

Prof. Taft *Dear Sir*—Having a perplexing case on hand, I desire a little information. About a year ago I extracted a superior right central and lateral incisors. The periosteum on both roots was destroyed by supuration. Thinking that the pus was carried off through the blood, I thought after the teeth were out all things would be right in a very short time. But not so. In about six months he returned; said that he had a gum boil just above where those teeth were taken out. I injected tepid water into the opening, cleaned it out thoroughly, then injected a solution of carbolic acid, iodine, and glycerine; gave him a bottle of it and a syringe; told him to use it every other day. It yielded to the treatment. I thought it was about well, and put up a partial set of teeth on rubber. In about two weeks he returned, saying it was getting worse. I advised him to lay the plate away, treat as before, and live *Hygienically*. I have used several other solutions, but to no effect. What can be done for the case? To-day he told me that it had discharged through the roof of the mouth, eighteen months after I took out the teeth. Now what shall I do with the case? what is the treatment indicated? I fear there may be something I do not recognize.

At your earliest convenience I shall be pleased to hear from you.

Very respectfully,

G. K.

This case is one in which there is without doubt considerable necrosis of the alveolus, especially of the inner plate; it has probably been extending since the removal of the teeth.

The systemic condition is not referred to, and we will presume it is good. The treatment then would consist in enlarging the fistulous canal, and the removal of the dead bone with the instruments, so far as possible; then treat with the elixir of vitrol to complete the work of removal of the necrosed bone. After this about all that will be necessary will be to mention the most favorable conditions for reparation, which may be accomplished by the use of a solution of carbolic acid, and tannin and glycerine.

All local irritants must be excluded, and the parts keep in rest as far as possible. It is far more difficult to prescribe from the description of another than from one's own examination.

### Expression of Appreciation.

The following token of appreciation we think eminently proper and justly due to one who is ever quick to see and prompt to act for the interest of that profession which he early learned to love, and has ever manifested a jealous care for its well-fare.

For the material things that tend to the growth and development of the dental profession, so far at least as its art and manipulative aspects are concerned, none can claim, nor are entitled to a more worthy and honorable mention than the subject of the following resolution. Ed.

RESOLUTION OF THANKS TO DR. S. S. WHITE, BY THE AMERICAN DENTAL ASSOCIATION, AT NIAGARA, AUG. 5, 1872.

Whereas, Dr. S. S. White, by his self-sacrificing and yet conservative action, has secured and preserved about all that seems to be left, in the way of evidence, against the validity of the "Cummings Patent," that may be employed with any hope of success, by the dental profession in a contest with the Dental Vulcanite Company.

The indications are that had he been free to act there was a possibility, and perhaps a strong probability, that a very different result would have been attained by the litigation; knowing as he did much of the duplicity and rascality of the plot, but yet not enough to warrant a public announcement.

For his efforts to save members of the profession from active cooperation in and money contribution to this swindle, he has received censure and recrimination from those he endeavored to save from defeat and involvement, therefore.

*Resolved*, That to him is due the thanks of this *Association*

for the course he has pursued, and the efforts he has made in this cause, which have an embodiment in the *expose* he has made of the whole scheme and transaction in the June number of the "*Dental Cosmos*." For all this he is entitled to and receives our entire approbation, appreciation, and regard.

### "Gold, Gold, Gold."

We are often asked "What gold do you prefer and are you using for filling." Well, we always use what we prefer, except in the way of experiment.

It is the duty of every dentist to try everything presented that promises any advantage or claims superiority in any respect. Every one should make such test for himself, then and then only can he know whether the claims made for any material or process will in his hands and experience be verified.

There is a lamentable want in the dental profession in this respect. A very large proportion of the members of the profession will not attempt to test any new thing that may be presented till others have tried it and found it valuable. This state of things results in a large measure from the want of a thorough professional education, with which is found an inability, to decide or form an opinion, upon any such matter by a knowledge of principles, or in any other way than by actual experiment.

There are others who will not give the time, money, and labor requisite to test new things, even when old ones have palpable defects, until they have been proven by others.

Now, in regard to gold, we have tried every form in which it has been presented, and have found almost every form to possess some desirable qualities. We have for several months past been using "Kearsings Prepared Foil Blocks," "Williams' Gold Cylinders," "Pack's Crystal Pellets," all the different numbers and sizes of each; and Williams' Nos. 60 and 30 Crystal Surface Foil. With all these at hand we can hardly see how anything else in form can be called for. These preparations seem to meet about all the requirements that gold is capable of fulfilling as a material for filling teeth.

